# *Listeria monocytogenes* Cytoplasmic Entry Induces Fetal Wastage by Disrupting Maternal Foxp3^+^ Regulatory T Cell-Sustained Fetal Tolerance

**DOI:** 10.1371/journal.ppat.1002873

**Published:** 2012-08-16

**Authors:** Jared H. Rowe, James M. Ertelt, Lijun Xin, Sing Sing Way

**Affiliations:** Departments of Pediatrics and Microbiology, Center for Infectious Disease and Microbiology Translational Research, University of Minnesota School of Medicine, Minneapolis, Minnesota, United States of America; Stanford University School of Medicine, United States of America

## Abstract

Although the intracellular bacterium *Listeria monocytogenes* has an established predilection for disseminated infection during pregnancy that often results in spontaneous abortion or stillbirth, the specific host-pathogen interaction that dictates these disastrous complications remain incompletely defined. Herein, we demonstrate systemic maternal *Listeria* infection during pregnancy fractures fetal tolerance and triggers fetal wastage in a dose-dependent fashion. *Listeria* was recovered from the majority of concepti after high-dose infection illustrating the potential for *in utero* invasion. Interestingly with reduced inocula, fetal wastage occurred without direct placental or fetal invasion, and instead paralleled reductions in maternal Foxp3^+^ regulatory T cell suppressive potency with reciprocal expansion and activation of maternal fetal-specific effector T cells. Using mutants lacking virulence determinants required for *in utero* invasion, we establish *Listeria* cytoplasmic entry is essential for disrupting fetal tolerance that triggers maternal T cell-mediated fetal resorption. Thus, infection-induced reductions in maternal Foxp3^+^ regulatory T cell suppression with ensuing disruptions in fetal tolerance play critical roles in pathogenesis of immune-mediated fetal wastage.

## Author Summary

Pregnant women are uniquely susceptible to the bacterium *Listeria monocytogenes* that preferentially lives inside infected cells. Since infection during pregnancy often triggers prematurity, abortion, or stillbirth, we propose that understanding how these complications occur represent important prerequisites for improving the health of mothers and the developing fetus. Here we investigate the specific interaction between *Listeria* and a subset of immune-suppressive cells in the mother that expand to create and maintain a hospitable environment for the fetus. We find although *Listeria*, especially with highdosage infection, can invade the fetus, damage can also occur by infection-induced changes in maternal immune cells that make it markedly less hospitable for the fetus. Under these circumstances, maternal immune-mediated rejection causes fetal injury without direct pathogen invasion. We show this occurs using both reduced-dosages of virulent or weakened *Listeria* for infection. By comparing pregnancy outcomes and infection-induced changes in the mother that make it more or less hospitable for the fetus, we further demonstrate Listeria gaining entry inside infected cells is the critical factor for immune-mediated fetal injury. These results illustrating infection-induced changes in the mother that lead to fetal injury have important implications for designing new ways to improve the outcomes of pregnancy.

## Introduction

*Listeria monocytogenes* (Lm) is a ubiquitous human pathogen with a unique predisposition for invasive disseminated infection during pregnancy that represents a significant etiology of spontaneous abortion, stillbirth, and neonatal infection [1], [2]. Although many Lm-specific proteins required for cell entry and maintaining residence within infected cells have been identified, and some play important roles in placental cell invasion [3], [4], the interplay between Lm and maternal immune cells that sustain fetal tolerance in the pathogenesis of infection-induced fetal injury has not been well-characterized. Recently, the physiological accumulation of immune suppressive maternal Foxp3^+^ regulatory CD4 T cells (Tregs) during gestation was shown to compromise host defense against Lm and other pathogens that cause prenatal infection [5]. Nevertheless, despite increased infection susceptibility, the sustained expansion of maternal Tregs was more essential because even transient partial ablation to baseline levels was sufficient to disrupt fetal tolerance and trigger fetal resorption [5], [6], [7]. These findings in mice recapitulate the blunted expansion of maternal Tregs with spontaneous abortion and other complications associated with fractured fetal tolerance in human pregnancy [8], [9], [10], [11], [12]. Thus, healthy pregnancy requires the sustained accumulation of immune-suppressive maternal Tregs that maintains tolerance to the developing fetus.

In addition to these quantitative changes, fluctuations in Treg suppressive potency also occur. These shifts fine-tune the delicate fluid balance between immune stimulation and suppression. In particular within the first few days after infection in non-pregnant mice with Lm or other pathogens that primarily cause acute infection, progressive reductions in either Treg number or their suppressive potency have each been described [13], [14]. Similarly, immune activation that coincides with pathogen clearance during more persistent infections occurs with either experimental Treg ablation or naturally occurring reductions in Treg suppressive potency [15], [16], [17], [18]. Accordingly, infection-induced reductions in Treg suppression are likely essential prerequisites for unleashing the activation of immune effectors that efficiently eradicate infection [19]. Importantly however, how pregnancy-expanded Tregs impact infection-induced shifts in suppression, and reciprocally how infection-induced shifts in maternal Treg suppression impact fetal tolerance are each undefined. Given the substantial overlap in pregnancy complications (e.g. spontaneous abortion, stillbirth, prematurity) associated with prenatal Lm infection and disruptions in fetal tolerance induced by experimental or naturally-occurring defects in maternal Treg accumulation, we sought to investigate if infection-induced shifts in maternal Treg suppression might disrupt fetal tolerance and cause fetal wastage. Immune-mediated fetal injury triggered by maternal infection that may occur without *in utero* pathogen invasion could explain the only modest fraction of concepti with recoverable Lm born to mothers with invasive infection [20], and spur new approaches for improving pregnancy outcomes.

To address these questions, we investigated maternal Tregs and the maintenance of fetal tolerance using escalating dosages of virulent Lm for infection during pregnancy. Foxp3^GFP^ reporter mice were used so that maternal Tregs could be purified based on their lineage-defining marker [21], and a mating strategy using ovalbumin (OVA)-expressing males allowed the maternal response to this surrogate fetal antigen to be precisely characterized [5], [22], [23]. To more specifically evaluate the contribution of immune-mediated fetal wastage in isolation, pregnancy outcomes, maternal Tregs, and fetal tolerance were also compared using Lm mutants lacking defined virulence determinants required for *in utero* invasion. Together, these studies demonstrate Lm entry into the cell cytoplasm disrupts fetal tolerance sustained by maternal Foxp3^+^ Tregs that triggers immune-mediated fetal wastage.

## Results

### *Listeria monocytogenes* prenatal infection induces dose-dependent fetal resorption

**Figure 1:**
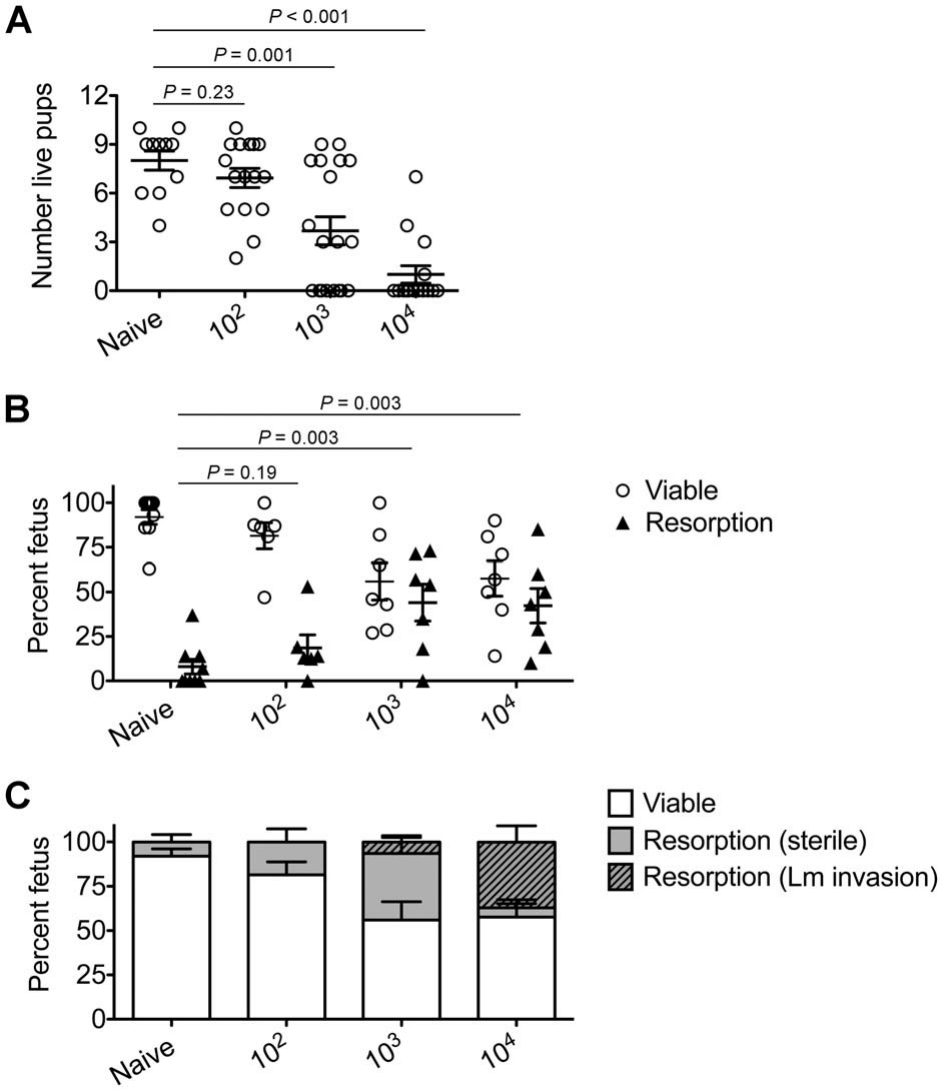
*Listeria monocytogenes* infection triggers dose-dependent rates of fetal wastage during allogeneic pregnancy. (A) Number of live pups born with virulent WT Lm inoculated midgestation (E10.5) at the indicated dosages for pregnant C57Bl/6 females mated with Balb/c males. (B) Percent viable and resorbed fetuses five days after WT Lm infection at midgestation. (C) Percent viable and resorbed fetuses with recoverable Lm for the mice described in B. Each data point represents results from an individual mouse combined from three independent experiments each with similar results.

To investigate the pathogenesis of prenatal listeriosis, pregnancy outcomes after infection with escalating dosages of virulent Lm beginning midgestation were evaluated. In this regard, although Lm has been described to stimulate fetal resorption and *in utero* invasion during syngeneic pregnancy [24], [25], [26], [27], this mating scheme does not recapitulate the natural heterogeneity between maternal and paternal antigens, and more pronounced accumulation of maternal Tregs [5], [6], [7]. To bypass these limitations, pregnancy outcomes were enumerated using MHC mismatched strains of inbred mice (Balb/c H-2^d^ males with C57Bl/6 H-2^b^ females) for mating. We found Lm infection midgestation (E10.5) caused dose-dependent reductions in the number of live pups born at term ∼10 days thereafter (Figure 1A). Compared with uninfected pregnancies [8.0±0.6 live pups (mean ± standard deviation)], the number of live pups was reduced by 88% and 54% [1.0±0.5 (10^4^ CFUs); 3.7±0.9 (10^3^ CFUs)] for mice infected with each respective Lm dosage (Figure 1A). Furthermore, no live pups were born for the majority of pregnant mice (9 of 13) infected with 10^4^ Lm. Thus, maternal Lm infection during allogeneic pregnancy in mice triggers fetal wastage.

To more comprehensively evaluate infection-induced fetal injury, the frequency of *in utero* fetal resorption at an earlier time point after infection (day 5 post-infection, E15.5) was also enumerated. Consistent with dose-dependent reductions in the number of live pups born at term, progressively increased rates of fetal wastage were found with escalating dosages of Lm used for infection (Figure 1B). Interestingly, by culturing each individual resorbed placental-fetal unit, dose-dependent increased rates of fetal Lm invasion that were uniformly reduced compared with the overall resorption frequencies were also identified. In particular, while the majority of resorbed fetuses (87%) contained recoverable Lm after infection with the highest inocula (10^4^ CFUs), the recovery of bacteria among resorbed fetuses declined sharply with reduced inocula (14% for 10^3^ CFUs, and 0% for 10^2^ CFUs) (Figure 1C). Given the previously described decline in recoverable bacteria in the placenta and fetus at earlier time points (from 24 through 72 hours) after maternal infection during syngeneic pregnancy [28], we also enumerated Lm at these time points following infection with the intermediate 10^3^ Lm dosage to investigate the possibility that the absence of recoverable bacteria in resorbed placental-fetal units could reflect Lm invasion that had been cleared by day 5. We found the placenta and fetus each contained no recoverable bacteria (among 26 and 16 individual placental-fetal units 24 and 72 hours, respectively, following maternal Lm infection midgestation) at these earlier time points (data not shown). Furthermore, the absence of direct Lm placental-fetal invasion for the majority of resorbed concepti using this intermediate 10^3^ Lm dosage was reinforced by the absence non-viable bacteria using PCR-based detection with primers specific for Lm *hly* and *lmo0056* among culture negative concepti (data not shown). Together, these results demonstrate although Lm has the potential for *in utero* invasion, especially after high-dose infection, infection-induced fetal injury can also occur without direct pathogen invasion of the placenta or fetal tissue.

### *Listeria monocytogenes* blunts maternal Treg suppression and disrupts fetal tolerance

**Figure 2:**
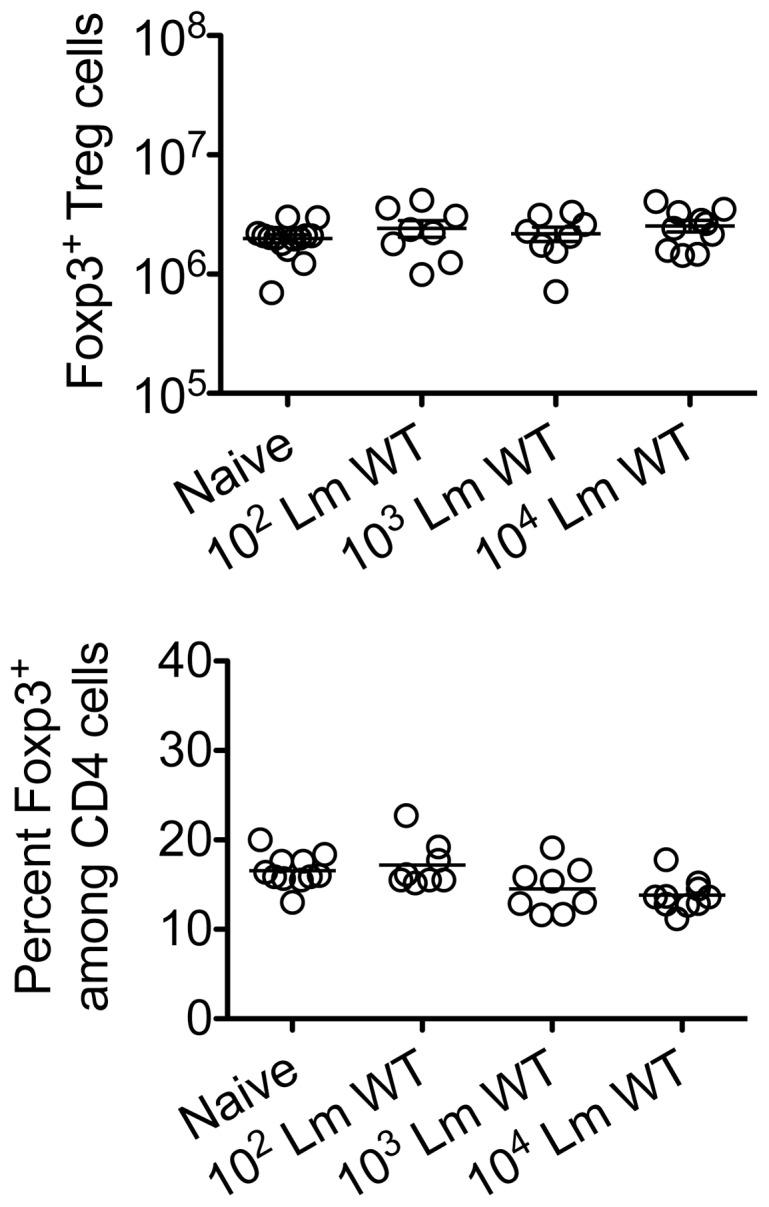
*Listeria monocytogenes* infection during pregnancy does not induce quantitative changes in maternal Foxp3^+^ regulatory CD4 T cells. Absolute number (top) and percent Foxp3^+^ among CD4 T cells (bottom) three days after virulent WT Lm infection at midgestation in C57Bl/6 females mated with Balb/c males. Each data point represents results from an individual mouse combined from two independent experiments each with similar results.

**Figure 3:**
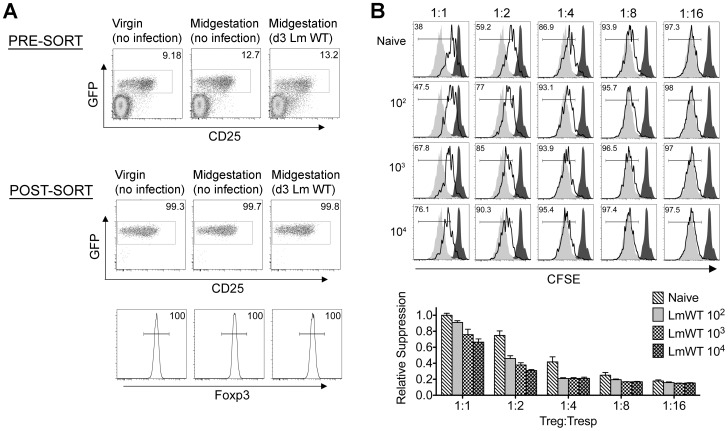
*Listeria monocytogenes* infection during pregnancy dampens maternal Foxp3^+^ regulatory CD4 T cell suppressive potency. (A) Percent GFP^+^ or Foxp3^+^ among CD4 cells in virgin, pregnant mice midgestation without infection, or three days after infection with 10^4^ WT Lm. (B) Representative plots demonstrating proliferation (CFSE dilution) among responder (Tresp) CD90.1^+^ CD8 cells after co-culture with each ratio of GFP^+^ Tregs isolated from Lm-infected mice and stimulation with anti-CD3 antibody (black line), compared with no Treg (gray filled) or no stimulation (black filled) controls (top). Relative suppression of responder cell proliferation (CFSE dilution) after co-culture with GFP^+^ Tregs for the mice described above normalized to suppression by GFP^+^ cells from uninfected controls at a 1∶1 Treg∶Tresp ratio (bottom). These data reflect six to eight mice per group representative of three independent experiments each with similar results.

Given the importance of expanded maternal Foxp3^+^ Tregs in maintaining pregnancy [5], [6], [7], and infection-induced reductions in Treg suppression that unleash immune activation in non-pregnant mice [13], [14], [17], [18], [19], we investigated if similar reductions in maternal Treg suppression occur with infection during pregnancy. We found Lm infection at midgestation did not significantly impact either the number or percent Foxp3^+^ among CD4 cells, suggesting quantitative reductions in maternal Tregs do not occur in this context (Figure 2). Next, to investigate the potential for infection-induced qualitative shifts in maternal Treg suppressive potency, Foxp3^GFP^ reporter mice on the C57Bl/6 background were substituted for mating with Balb/c males so that maternal Foxp3^+^ Tregs could be purified as GFP^+^ CD4 cells by FACS directly *ex vivo*. Consistent with prior studies in non-pregnant mice [14], [21], maternal Tregs were isolated from pregnant mice with equally high purity by sorting for GFP^+^ CD4 cells (Figure 3A). By enumerating the efficiency whereby these GFP^+^ CD4 cells suppress the proliferation of responder T cells in co-culture, no significant difference in suppressive potency were found for Tregs recovered from pregnant compared with non-pregnant control mice (Figure S1 in Text S1).

By contrast with escalating Lm dosages used for infection beginning midgestation, progressive reductions in maternal Treg suppressive potency were identified because the proliferation of responder T cells isolated from naïve mice was more pronounced after co-culture with maternal GFP^+^ Tregs from infected compared with uninfected control mice (Figure 3B). To evaluate the magnitude of these infection-induced reductions in Treg suppressive potency, we titrated the ratio of GFP^+^ Tregs to responder T cells in co-culture and found two-fold increased ratios of Tregs from pregnant mice infected with 10^3^ Lm were required to achieve the same level of suppression as Tregs cells recovered from uninfected mice (Figure 3B). Comparatively, GFP^+^ Tregs recovered from mice infected with 10^4^ Lm suppressed responder cell proliferation even less efficiently; requiring two- to four-fold more Tregs to achieve the same level of suppression compared with GFP^+^ Tregs recovered from uninfected controls, while Tregs recovered from mice infected with 10^2^ Lm suppressed responder cell proliferation to an intermediate degree compared with Tregs isolated from uninfected mice and those infected with higher Lm inocula (Figure 3B). Thus, Lm infection during pregnancy stimulates dose-dependent reductions in maternal Treg suppressive potency comparable to reductions in Foxp3^+^ CD4 cell suppressive potency with acute systemic Lm infection in non-pregnant mice [14].

**Figure 4:**
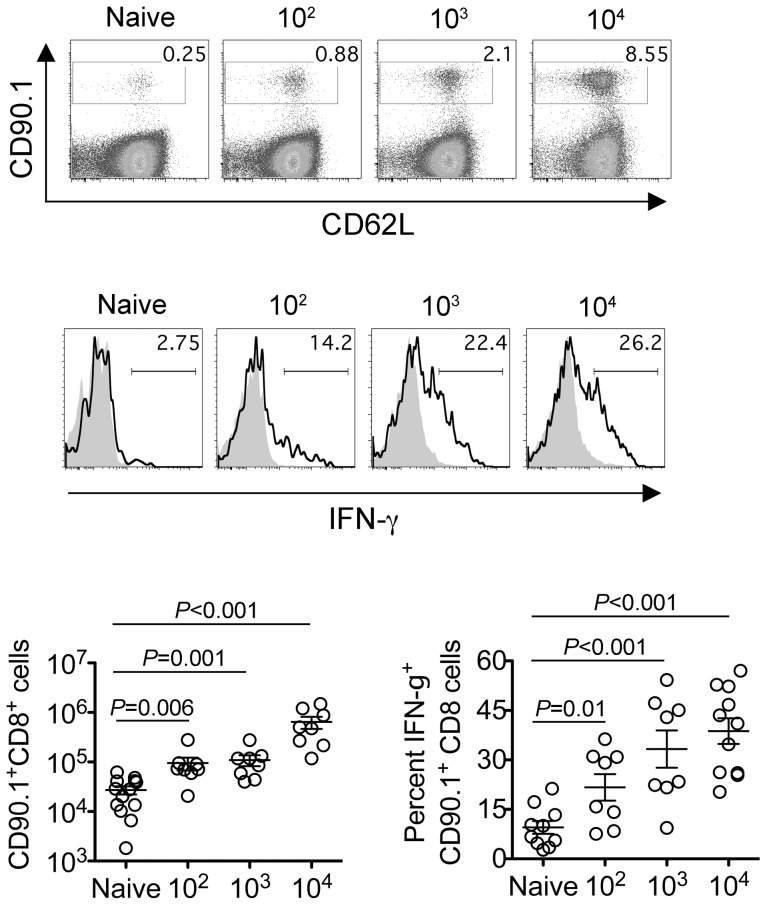
*Listeria monocytogenes* infection during pregnancy disrupts fetal tolerance. Representative FACS plots (top) and composite data (bottom) illustrating expansion and IFN-γ production by fetal-OVA-specific CD8 T cells among maternal splenocytes in mice impregnated by Actin-OVA males five days after Lm infection at midgestation. For IFN-γ production, cells were stimulated with OVA_257–264_ peptide (black line) or no peptide controls (gray filled). Each data point represents results from an individual mouse combined from three independent experiments each with similar results.

Since the sustained expansion of maternal Foxp3^+^ Tregs is essential for maintaining tolerance to paternal antigens expressed by the developing fetus [5], [6], [7], we further investigated how these infection-induced reductions in maternal Treg suppressive potency might impact fetal tolerance. To identify maternal cells with fetal specificity, transgenic male mice engineered to express the model antigen, OVA, in all cells behind the β-actin promoter were substituted for mating with C57Bl/6 females so that maternal T cells responsive to peptides within the surrogate fetal-OVA antigen can be tracked using established immunological tools [5], [22], [23]. Following Lm infection at midgestation, we found maternal CD8 T cells with fetal-OVA specificity accumulated and became activated in a dose-dependent fashion (Figure 4). Specifically, compared with the few OVA-specific cells recovered from uninfected pregnant mice that produced only background levels of IFN-γ, fetal-OVA-specific T cells expanded over 50-fold and efficiently produced IFN-γ in pregnant mice infected with 10^4^ Lm (Figure 4). Although the degree of expansion and cytokine production each progressively diminished with reduced Lm inocula, both remained significantly elevated compared with background levels found in uninfected pregnant mice. Together, these results demonstrate Lm infection during pregnancy blunts maternal Treg suppression and disrupts fetal tolerance. Furthermore, since even transient partial reductions in maternal Treg numbers cause resorption and fractures fetal tolerance [5], fetal wastage that occurs without direct invasion of the placental-fetal unit with lower Lm inocula are likely triggered by infection-induced reductions in maternal Treg suppressive potency.

### Cytoplasmic entry is essential for *Listeria monocytogenes*-induced fetal resorption

**Figure 5:**
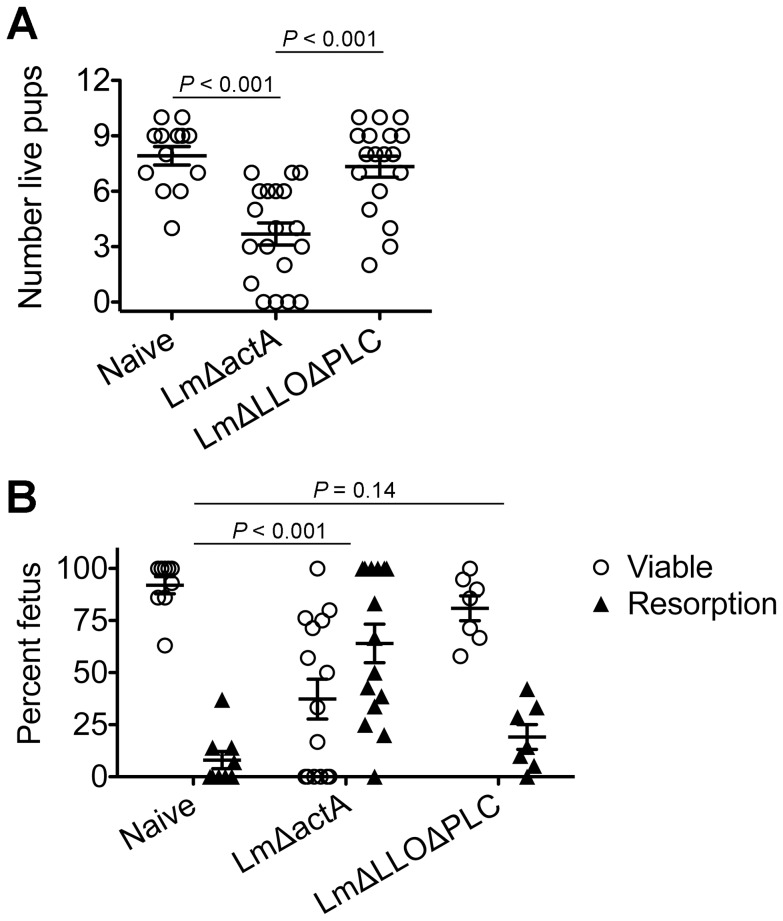
Cytoplasmic entry is essential for *Listeria monocytogenes*-induced fetal wastage. (A) Number of live pups born with LmΔactA (10^7^ CFUs) or LmΔLLOΔPLC (10^8^ CFUs) infection at midgestation (E10.5) in C57Bl/6 females mated with Balb/c males. (B) Percent viable and resorbed fetuses five days after LmΔactA (10^7^ CFUs) or LmΔLLOΔPLC (10^8^ CFUs) infection at midgestation. Each data point represents results from an individual mouse combined from three independent experiments each with similar results.

To more specifically investigate the pathogenesis of immune-mediated fetal injury that occurs with infection-induced disruption in maternal-fetal tolerance, we compared pregnancy outcomes after infection with attenuated Lm containing defects in defined virulence determinants required for productive infection that do not cause fetal invasion [3], [4], [26]. These include LmΔLLOΔPLC that cannot escape from the endocytic vacuole and enter into the cell cytoplasm; and LmΔactA that enters the cell cytoplasm, but cannot recruit actin required for intra- and inter-cellular spread [29]. In particular, these mutants were chosen because their ability to stimulate protective T cells *in vivo* that requires overriding Treg suppression is drastically discordant; LmΔactA readily primes the expansion of protective T cells, whereas LmΔLLOΔPLC does not [30], [31], [32]. Consistent with robust immune activation that occurs with Lm entry into the cell cytoplasm [33], LmΔactA infection midgestation caused sharp reductions in the number of live pups with reciprocal increased rates of fetal resorption compared with uninfected controls (Figure 5A and B). By contrast, the number of live pups and frequency of fetal resorption did not differ significantly between pregnant mice infected with LmΔLLOΔPLC and non-infected controls (Figure 5A and B). Importantly, these differences in pregnancy outcomes could not be attributed to potential differences in relative attenuation between these two Lm strains because 10-fold more LmΔLLOΔPLC compared with LmΔactA was used for infection, and at these dosages no significant difference in bacterial CFUs were found one day post-infection in the liver representing another tissue susceptible to Lm invasion (Figure S2 in Text S1). Furthermore, consistent with the highly attenuated nature of these mutants, LmΔactA and LmΔLLOΔPLC each became eradicated from the liver, and could not be recovered from any resorbed placental-fetal units by day 5 post-infection (data not shown). Together, these results demonstrate Lm cytoplasmic entry is essential for infection-induced fetal wastage.

### *Listeria monocytogenes* cytoplasmic entry blunts maternal Treg suppression and disrupts fetal tolerance

**Figure 6:**
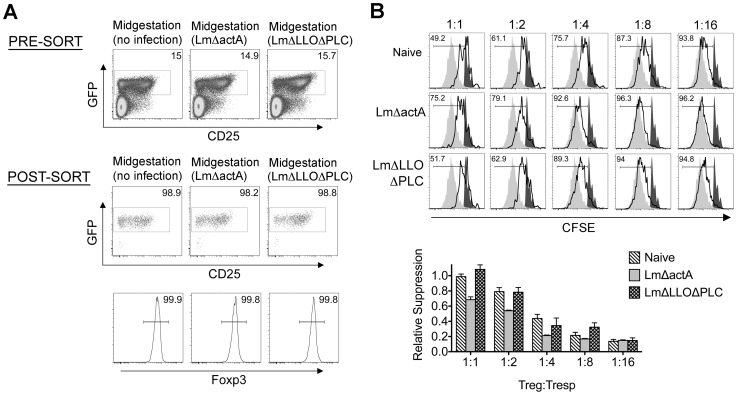
*Listeria monocytogenes* cytoplasmic entry dampens maternal Foxp3^+^ regulatory CD4 T cell suppressive potency. (A) Percent GFP^+^ or Foxp3^+^ among CD4 cells in pregnant mice midgestation after infection with LmΔactA (10^7^ CFUs) or LmΔLLOΔPLC (10^8^ CFUs). (B) Representative plots demonstrating proliferation (CFSE dilution) among responder (Tresp) CD90.1^+^ CD8 cells after co-culture with each ratio of GFP^+^ Tregs isolated from LmΔactA (10^7^ CFUs) or LmΔLLOΔPLC (10^8^ CFUs) infected mice, and stimulation with anti-CD3 antibody (black line), compared with no Treg (gray filled) or no stimulation (black filled) controls (top). Relative suppression of responder cell proliferation (CFSE dilution) after co-culture with GFP^+^ Tregs for cells from mice described above normalized to suppression by GFP^+^ cells from uninfected controls at a 1∶1 Treg∶Tresp ratio (bottom). These data reflect six to eight mice per group representative of three independent experiments each with similar results.

Given reductions in Treg suppressive potency associated with fetal resorption and fractured fetal tolerance after virulent Lm infection (Figures 1, 3, and 4), we investigated if differences in fetal wastage induced by LmΔactA and LmΔLLOΔPLC also paralleled discordance efficiencies in dampening maternal Treg suppression and disrupting fetal tolerance. GFP^+^ Tregs could be purified efficiently from pregnant Foxp3^GFP^ reporter mice after LmΔactA and LmΔLLOΔPLC infection similar to mice after WT Lm infection or uninfected controls (Figure 6A). Using purified GFP^+^ Tregs, we found LmΔactA infection midgestation triggered ∼2-fold reductions in suppressive potency for maternal Tregs compared with GFP^+^ CD4 cells recovered from uninfected pregnant controls (Figure 6B). Interestingly, the magnitude of these reductions in Treg suppressive potency were almost identical to cells recovered from mice infected with low or intermediate WT Lm dosages that induce fetal resorption with minimal to undetectable *in utero* invasion. By contrast, the suppressive potency for maternal GFP^+^ Tregs after LmΔLLOΔPLC infection did not differ significantly compared with cells from uninfected controls (Figure 6B). Thus, reductions in maternal Treg suppressive potency directly parallel fetal wastage induced by LmΔactA and LmΔLLOΔPLC.

**Figure 7:**
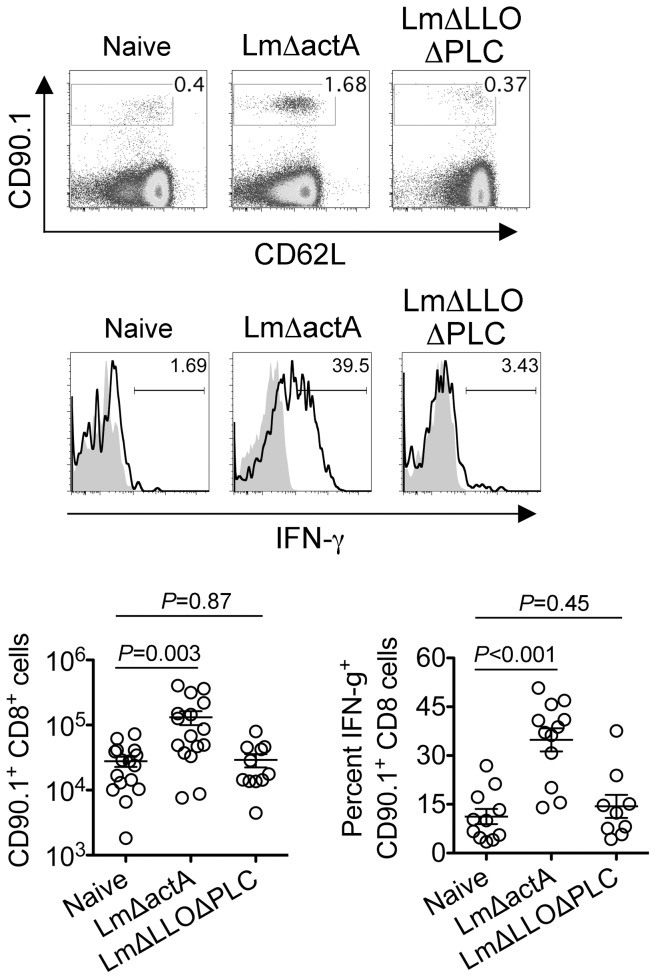
*Listeria monocytogenes* cytoplasmic entry disrupts fetal tolerance with infection during pregnancy. Representative FACS plots (top) and composite data (bottom) illustrating expansion and IFN-γ production by fetal-OVA-specific CD8 T cells among maternal splenocytes in mice impregnated by Actin-OVA males five days after LmΔactA (10^7^ CFUs) or LmΔLLOΔPLC (10^8^ CFUs) infection at midgestation. For IFN-γ production, cells were stimulated with OVA_257–264_ peptide (black line) or no peptide controls (gray filled). Each data point represents results from an individual mouse combined from three independent experiments each with similar results.

To further establish how these reductions in maternal Treg suppression induced by attenuated Lm impact fetal tolerance, the expansion and activation of maternal T cells with specificity to the surrogate fetal-OVA antigen were also enumerated after infection in pregnant C57Bl/6 mice mated with OVA-expressing Balb/c males. Similar to WT Lm, LmΔactA inoculated midgestation primed the robust expansion and IFN-γ production by fetal-OVA specific T cells (Figure 7). Comparatively, LmΔLLOΔPLC failed to stimulate expansion and IFN-γ production above background levels found in uninfected control mice. These findings directly parallel the relative efficiency whereby each attenuated Lm dampens maternal Treg suppressive potency and induces fetal wastage (Figures 5 and 6). Lastly, to more definitively establish infection-induced immune-mediated fetal injury, the impacts of maternal CD4 and CD8 T cell depletion prior to Lm infection on pregnancy outcomes were evaluated. These experiments exploit the highly attenuated nature of LmΔactA that is eliminated even in immune-compromised mice to investigate how depletion of effector and regulatory T cells together impact infection-induced fetal wastage. Remarkably, the rate of LmΔactA-induced fetal wastage became sharply reduced in T cell-depleted compared with T cell-sufficient pregnancies [11.2±3.5% resorption in T cell depleted mice (n = 9); 62.7±9.5% resorption in T cell-sufficient mice (n = 15), *P* = 0.0005]. Reciprocally, the number of live pups born with LmΔactA infection initiated midgestation was significantly increased in T cell-ablated compared with T cell sufficient pregnancies [5.80±0.49 live pups in T cell depleted mice (n = 10) compared with 3.68±0.60 live pups in T cell-sufficient pregnancies (n = 19), *P* = 0.027]. Taken together, these results demonstrate Lm cytoplasmic entry is essential for disrupting fetal tolerance that triggers maternal T cell-mediated fetal wastage.

## Discussion

The intracellular bacterium Lm represents a significant infectious cause of pregnancy loss and stillbirth [1], [2]. Herein, we investigate the host pathogen interaction that triggers these unfortunate outcomes, with particular focus on how prenatal infection impacts fetal tolerance sustained by expanded maternal Foxp3^+^ Tregs. Our results demonstrate infection-induced dampening of Treg suppression that unleashes immune activation required for optimal host defense [19], in the context of prenatal infection when sustained tolerance to fetal antigen is essential, plays a pivotal role in the “immune-pathogenesis” of fetal wastage. The importance of immune-mediated fetal injury is shown by the increased frequency of fetal resorption with reciprocal reduction in the number of live pups following infection with low or intermediate doses of virulent Lm where bacteria are not found in the majority of resorbed concepti. Similarly for attenuated Lm that do not cause fetal invasion [26], fetal wastage and disrupted fetal tolerance occurs only for strains that retain the ability to prime protective T cells through entry into the cell cytoplasm and dampen maternal Treg suppressive potency. Furthermore, depletion of effector T cells along with Tregs prior to cytoplasmic Lm infection sharply reduces the frequency of infection-induced fetal wastage. Together with fetal injury induced by systemic treatment with various TLR ligands [e.g. LPS, poly(I∶C)] shown to modulate Treg suppression *in vitro* or after *in vivo* stimulation in non-pregnant mice [34], [35], [36], [37], [38], [39], these results establish the importance of infection- or inflammation-induced disruption of maternal Treg suppression in the pathogenesis of fetal wastage. Although we used intravenous inoculation to recapitulate disseminated infection that occurs with Lm during pregnancy, crucial next steps based on these results are to evaluate if systemic disruption of fetal tolerance is essential, or if local disruption induced by pathogens that primarily reside in the vaginal or cervical mucosa (e.g. bacterial vaginosis, *Ureaplasma* and *Chlamydia* sp.) are also sufficient to stimulate fetal injury.

**Figure 8:**
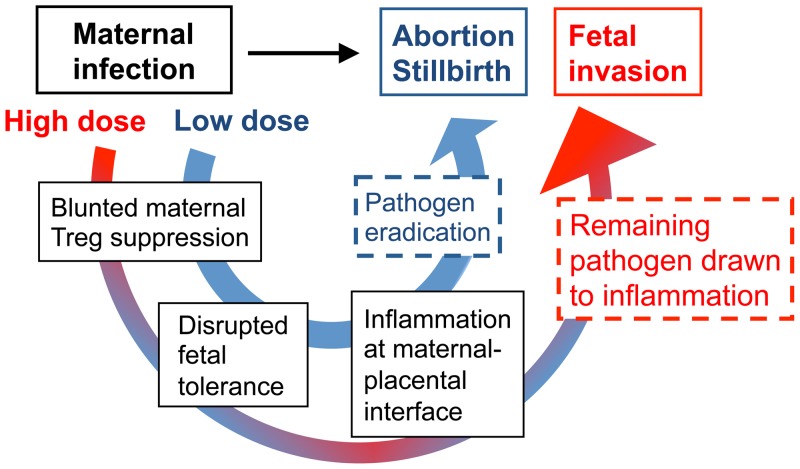
Proposed model for immune-mediated fetal wastage induced by prenatal infection that can occur with or without *in utero* pathogen invasion. After low dose infection during pregnancy, reductions in maternal regulatory T cell suppression unleash the activation of immune effectors enough to rapidly eliminate the pathogen. However, given the requirement for sustained expansion of maternal regulatory cell suppression in maintaining fetal tolerance, these reductions in suppressive potency also trigger immune-mediated fetal wastage. By comparison with higher dosage infection, blunted maternal regulatory T cell suppression that promotes immune activation does not eradicate infection as efficiently. In turn with ongoing disruption in fetal tolerance, remaining pathogen is drawn to inflammation at the uterine-placental interface that promotes invasion into the placental-fetal unit.

On the other hand, since Lm and other prenatal pathogens can and do cause *in utero* fetal invasion, immune-mediated fetal injury alone does not fully address the pathogenesis of these infections. In this regard, an important clue from our studies is that although virulent Lm-induced fetal resorption and invasion are each dose-dependent, these processes can be readily dissociated based on the inocula of Lm used for infection. Accordingly, we propose a model whereby low-dose maternal infection dampens Treg suppression enough to stimulate the activation of immune effectors that rapidly eliminate the pathogen so that fetal injury occurs almost exclusively via immune-mediated pathways (Figure 8). Comparatively, with higher-dose infection, blunted maternal Treg suppression that promotes immune activation does not eradicate infection as efficiently. In turn with ongoing disruptions in fetal tolerance, remaining pathogen drawn to inflammation at the uterine-placental interface promotes invasion into the placental-fetal unit (Figure 8). Although clearly an over-simplification, this model suggests overriding maternal Treg suppression is the pivotal feature that dictates whether fetal wastage occurs regardless of *in utero* pathogen invasion.

Together with prior studies using pregnant guinea pigs where the placental anatomy more closely resembles human tissue that illustrate the placenta is relatively resistant to early infection [40], [41], our findings suggest Lm infection-induced disruption of fetal tolerance with ensuing inflammation at the maternal-placental interface likely represents an important initial step in targeting bacteria for placental invasion. Later after infection when the placenta becomes a possible nidus for ongoing bacterial dissemination, expulsion or resorption of the placental-fetal unit by maternal immune effectors unleashed for activation by reduced Treg suppression provides expanded protection to the mother from infection at the expense of fetal injury [40], [41]. Based on these results, important areas for further investigation are to identify the cellular receptors and secreted cytokines that respond to Lm, and in particular bacteria in the cell cytoplasm, that stimulate reductions in maternal Treg suppression. Given the cellular immunology and transgenic tools that are currently available only in mice, our ongoing studies continue to address these questions experimentally using murine pregnancy models. However, additional investment in developing more refined immunological tools in other complementary animal models will be important for extending this work to more fully unravel the mechanistic steps in fetal wastage triggered by prenatal infection.

Finally, given the increasingly established heterogeneity and functional specialization among Foxp3^+^ CD4 cells that utilize distinct cell-associated and secreted molecules to mediate context specific immune suppression [42], [43], [44], establishing the Treg-associated molecule(s) that sustain fetal tolerance and distinguishing them from those required for host defense against infection have salient implications for developing therapies for dissociating the beneficial and detrimental impacts of expanded maternal Tregs. In this regard, while IL-10 is non-essential for sustaining fetal tolerance under non-inflammatory conditions, it likely plays more important roles in maintaining pregnancy under inflammatory conditions known to trigger fetal wastage [5], [35], [45], [46]. Accordingly, establishing the importance of other Treg-associated molecules in maintaining fetal tolerance and sustaining pregnancy represent other important areas for future investigation. Given the sharply increased rates of fetal wastage and pregnancy loss that occurs with prenatal Lm infection, the specific Treg-associated molecules essential for maintaining pregnancy may overlap with those required for optimal protection against prenatal infection. Nevertheless, given the importance of maternal Tregs in both sustaining fetal tolerance and compromising host defense against prenatal infection, we propose establishing how these cells work in each context represent critical next steps towards new therapeutic approaches for improving pregnancy outcomes.

## Materials and Methods

### Ethics statement

This study was carried out in accordance with recommendations in the Guide for the Care and Use of Laboratory Animals of the National Institutes of Health. These specific protocols were approved by the University of Minnesota Institutional Animal Care and Use Committee (Animal Welfare Assurance Number A3456-01).

### Mice

C57Bl/6 (H-2^b^), Balb/c (H-2^d^), and B6.PL-*Thy1* (CD90.1) mice were purchased from The National Cancer Institute or The Jackson Laboratory. Foxp3^GFP^ mice backcrossed to C57Bl/6 mice, OVA-expressing backcrossed to Balb/c mice, and OT-I TCR transgenic mice maintained on a CD90.1 background have been described [21], [22], [23], [47]. The timing of pregnancy was determined by visualization of a copulation plug (embryonic day 0.5) after introducing virgin female with male mice.

### Infections

Lm strains 10403 s (WT), 1942 (ΔactA), and 2319 (ΔLLOΔPLC) were each grown to early log phase (OD_600_ 0.1) in brain heart infusion media at 37°C, washed and diluted with saline to 200 µl, and injected intravenously via the lateral tail vein [29], [31], [32]. The inoculum was verified for each infection by plating serial dilutions onto agar plates. For enumerating recoverable Lm CFUs, each placental-fetal unit or the liver was individually dissected, homogenized in saline containing 0.05% Triton X and cultured onto agar plates as described [5]. For enumerating non-viable Lm by PCR, DNA was extracted from each placental-fetal unit after homogenization, phenol chloroform extraction, and ethanol precipitation, and used as template DNA with primers specific for Lm *hly* and *lmo0056* [hly, 5′-TGATTCACTGTAAGCCATTTC-3′ and 5′-AGCACCACCAGCATCTCCGC-3′; *lmo0056*, 5′-CCAAGCGAACTACGTGATCG-3′ and 5′-TGCTCTTCTACTGCGTTTGC-3′] [48].

### Antibodies, flow cytometry, and cell transfer

Fluorophore-conjugated antibodies and other reagents for cell surface, intracellular cytokine, and intranuclear Foxp3 staining were purchased from eBioscience or BD Biosciences. The expansion of fetal-OVA-specific CD8 cells among splenocytes, and cytokine production after stimulating splenocytes with OVA_257–264_ peptide in media containing GolgiPlug (BD Biosciences) was enumerated using previously described procedures [5]. Specifically, for cell transfers, purified CD8^+^ cells (10^5^) isolated from OT-I TCR transgenic (CD90.1) mice were injected intravenously into recipient (CD90.2) mice one day prior to Lm infection at midgestation. For T cell depletion, purified anti-mouse CD4 (GK1.5) and anti-mouse CD8 (2.43) antibodies (BioXcell) were administered intraperitoneally (500 µg each antibody per mouse) one day prior to LmΔactA infection at midgestation.

### Treg suppression assay

For enumerating Treg suppressive potency, CD4 cells were first enriched by negative selection (Miltenyi Biotec) from Foxp3^GFP^ reporter mice, followed by sorting for the GFP^+^ CD4 subset [21]. In each experiment, GFP^+^ Tregs were verified to be >98% pure by staining for Foxp3 expression. Responder CD8^+^ T cells isolated from naïve CD90.1 mice were labeled with CFSE (5 µM for 10 minutes at room temperature), and co-cultured in triplicate in 96-well round bottom plates (1×10^4^ responder cells per well) with purified GFP^+^ Tregs at the indicated ratios. The relative suppressive potency of Tregs in each experiment was calculated by comparing responder cell proliferation (CFSE dilution) after co-culture with GFP^+^ Tregs from uninfected control mice as described [14], [18].

### Statistical analysis

The number of live pups, resorbed concepti, cell numbers, and percent cytokine producing cells were first analyzed and found to be normally distributed. Thereafter, differences between groups were analyzed using an unpaired Student's t test (Prism, Graph Pad) with *P*<0.05 taken as statistical significance.

## Supporting Information

Text S1 **Text S1 contains Figures S1 and S2. Pregnancy does not induce shifts in maternal regulatory CD4 T cell suppressive potency (Figure S1).** Representative plots demonstrating proliferation (CFSE dilution) among responder CD90.1^+^ CD8 cells (Tresp) after co-culture with each ratio of GFP^+^ Tregs isolated from virgin or pregnant C57Bl/6 mice midgestation after mating with Balb/c males, and stimulation with anti-CD3 antibody (black line), compared with no Treg (gray filled) or no stimulation (black filled) controls (top). Relative suppression of responder cell proliferation (CFSE dilution) after co-culture with GFP^+^ Tregs for the mice described above normalized to suppression by GFP^+^ cells from non-pregnant controls at a 1∶1 Treg∶Tresp ratio (bottom). **Comparable** ***in vivo*** **bacterial burden after LmΔactA and LmΔLLOΔPLC infection (Figure S2).** Number of recoverable CFUs in the liver one day after infection with 10^7^ LmΔactA compared with 10^8^ LmΔLLOΔPLC. Each data point represents results from an individual mouse from two independent experiments each with similar results.
(DOC)
