# Supplementary material for: Listeria monocytogenes Cytoplasmic Entry Induces Fetal Wastage by Disrupting Maternal Foxp3+ Regulatory T Cell-Sustained Fetal Tolerance
Source: PLoS Pathog. 2012 Aug 16;8(8):e1002873. doi: 10.1371/journal.ppat.1002873 (PMC3420962; doi:10.1371/journal.ppat.1002873)
Supplement: Text S1 — contains Figures S1 and S2. Pregnancy does not induce shifts in maternal regulatory CD4 T cell suppressive potency (Figure S1). Representative plots demonstrating proliferation (CFSE dilution) among responder CD90.1+ CD8 cells (Tresp) after co-culture with each ratio of GFP+ Tregs isolated from virgin or pregnant C57Bl/6 mice midgestation after mating with Balb/c males, and stimulation with anti-CD3 antibody (black line), compared with no Treg (gray filled) or no stimulation (black filled) controls (top). Relative suppression of responder cell proliferation (CFSE dilution) after co-culture with GFP+ Tregs for the mice described above normalized to suppression by GFP+ cells from non-pregnant controls at a 1∶1 Treg∶Tresp ratio (bottom). Comparable in vivo bacterial burden after LmΔactA and LmΔLLOΔPLC infection (Figure S2). Number of recoverable CFUs in the liver one day after infection with 107 LmΔactA compared with 108 LmΔLLOΔPLC. Each data point represents results from an individual mouse from two independent experiments each with similar results. (DOC) [file ppat.1002873.s001.doc]

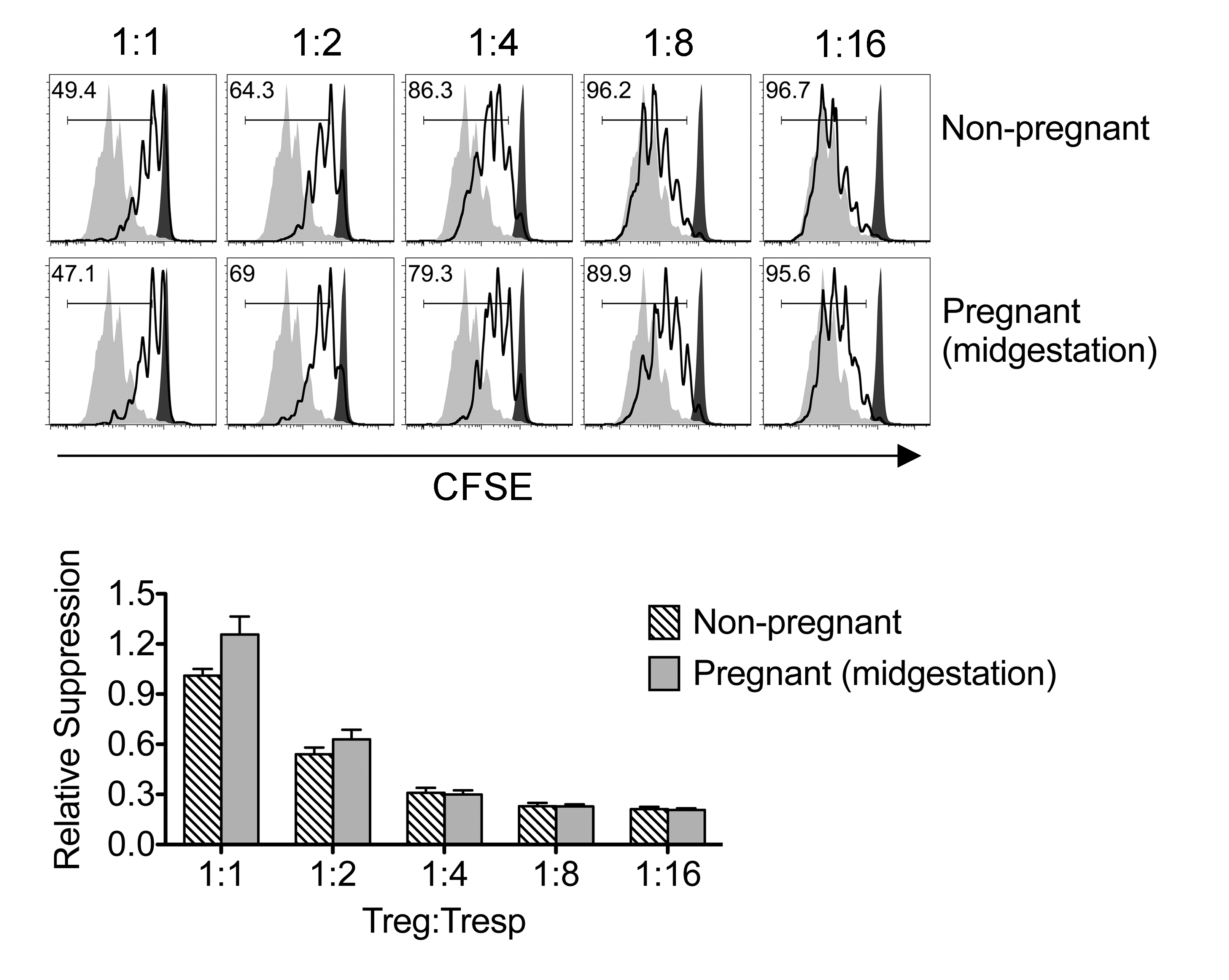


**Figure S1. Pregnancy does not induce shifts in maternal regulatory CD4 T cell suppressive potency.** Representative plots demonstrating proliferation (CFSE dilution) among responder CD90.1^+^ CD8 cells (Tresp) after co-culture with each ratio of GFP^+^ Tregs isolated from virgin or pregnant C57Bl/6 mice midgestation after mating with Balb/c males, and stimulation with anti-CD3 antibody (black line), compared with no Treg (gray filled) or no stimulation (black filled) controls (top). Relative suppression of responder cell proliferation (CFSE dilution) after co-culture with GFP^+^ Tregs for the mice described above normalized to suppression by GFP^+^ cells from non-pregnant controls at a 1:1 Treg:Tresp ratio (bottom).

**
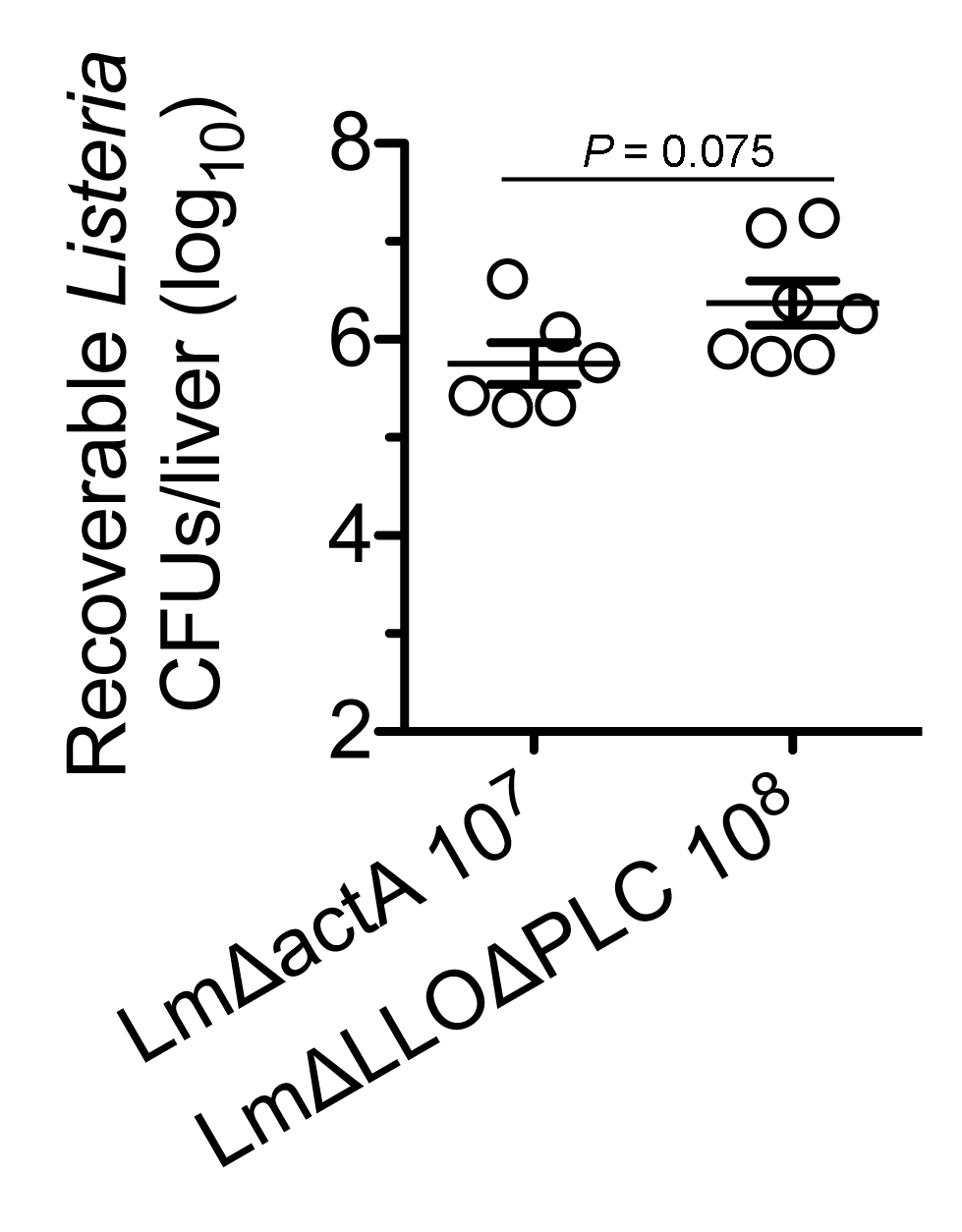
**

**Figure S2. Comparable *in vivo* bacterial burden after LmΔactA and LmΔLLOΔPLC infection.** Number of recoverable CFUs in the liver one-day after infection with 10^7^ LmΔactA compared with 10^8^ LmΔLLOΔPLC. Each data point represents results from an individual mouse from two independent experiments each with similar results.
